# Comparing treatment effects of oral THC on simulated and on-the-road driving performance: testing the validity of driving simulator drug research

**DOI:** 10.1007/s00213-015-3927-9

**Published:** 2015-05-10

**Authors:** J. L. Veldstra, W. M. Bosker, D. de Waard, J. G. Ramaekers, K. A. Brookhuis

**Affiliations:** Faculty of Behavioural and Social Sciences, University of Groningen, Grote Kruisstraat 2/1, 9712TS Groningen, The Netherlands; Institut für Neurowissenschaften und Medizin (INM-4), Forschungszentrum Jülich, 52425 Jülich, Germany; Faculty of Psychology and Neuroscience, Maastricht University, Universiteitssingel 40, 6229 ER Maastricht, The Netherlands

**Keywords:** Driving simulator, Driving performance, Dronabinol, Equivalence testing, Predictive validity, THC

## Abstract

**Rationale:**

The driving simulator provides a safe and controlled environment for testing driving behaviour efficiently. The question is whether it is sensitive to detect drug-induced effects.

**Objective:**

The primary aim of the current study was to investigate the sensitivity of the driving simulator for detecting drug effects. As a case in point, we investigated the dose-related effects of oral ∆^9^-tetrahydrocannabinol (THC), i.e. dronabinol, on simulator and on-the-road driving performance in equally demanding driving tasks.

**Method:**

Twenty-four experienced driver participants were treated with dronabinol (Marinol®; 10 and 20 mg) and placebo. Dose-related effects of the drug on the ability to keep a vehicle in lane (weaving) and to follow the speed changes of a lead car (car following) were compared within subjects for on-the-road versus in-simulator driving. Additionally, the outcomes of equivalence testing to alcohol-induced effects were investigated.

**Results:**

Treatment effects found on weaving when driving in the simulator were comparable to treatment effects found when driving on the road. The effect after 10 mg dronabinol was however less strong in the simulator than on the road and inter-individual variance seemed higher in the simulator. There was, however, a differential treatment effect of dronabinol on reactions to speed changes of a lead car (car following) when driving on the road versus when driving in the simulator.

**Conclusion:**

The driving simulator was proven to be sensitive for demonstrating dronabinol-induced effects particularly at higher doses. Treatment effects of dronabinol on weaving were comparable with driving on the road but inter-individual variability seemed higher in the simulator than on the road which may have potential effects on the clinical inferences made from simulator driving. Car following on the road and in the simulator were, however, not comparable.

## Introduction

The driving simulator is widely used to study driving behaviour (Wachtel 1995) mostly because it provides a safe and relatively controlled way of testing driving behaviour efficiently. This means that in the simulator we can safely investigate potential risks to traffic safety that are not safe to test on the road, for example, impaired driving as a consequence of the intake of medicines, drugs or alcohol. This is important because studies on the influence of such substances on driving performance can generate knowledge about fitness to drive after using these substances. Even so, the usability of the driving simulator for testing fitness to drive depends on its ability to elicit behaviour in the virtual environment that would also be displayed in the real world. In other words, the usability of the driving simulator as a means of investigating drug-induced effects on driving performance depends on its predictive validity, and it is this predictive validity that is often questioned (see for example Mullen et al. 2012 in Fisher et al. 2011).

The questions about the predictive validity of the driving simulator are usually two threaded (Fisher et al. 2011). If the numerical values between the naturalistic and the simulator data are equivalent, then ‘absolute’ validity can be claimed (Blaauw 1982; Godley et al. 2002). However, for a simulator to be a valid instrument for research, absolute validity is not essential (Törnros 1998). Research questions usually deal with matters relating to the effects of independent variables on dependent variables, rather than seeking to determine absolute numerical measurements of driver behaviour. Therefore, a second approach is to establish whether the same trend of effect is found for the driving simulator and on the road tests, assessing relative validity (Blaauw 1982; Godley et al. 2002).

Previous validation studies have usually investigated a specific task to determine if the simulator was a valid instrument of measurement in that specific case. Examples of these are studies on tasks such as keeping speed (Blaauw 1982; Törnros 1998; Bella 2008; Shinar and Ronen 2007) or lateral control (Blaauw 1982; Törnros 1998), braking (McGehee et al. 2000; Hoffman et al. 2002), responding to road markings (Godley et al. 2002) and studies on driver errors (Shechtman et al. 2009). These studies generally have reported that driving in the simulator corresponds fairly well to on-road driving (see also Fisher et al. 2011). Even so, Volkerts et al. (1992) questioned whether the driving simulator would also be sensitive enough to detect drug-induced effects.

Generally, driving simulator studies investigating the effects of different types of drugs and alcohol on driving performance show a similar trend of effect in the simulator as are also reported in on-the-road studies. For example, in two driving simulator studies conducted by Mets et al. (2011) and Veldstra et al. (2011), the same dose-dependent relationship between alcohol and standard deviation of the lateral position (SDLP) was reported that was also reported in an on-the-road driving study by Louwerens et al. (1987). Direct within-subjects comparisons between drug-induced effects in simulated and on-the-road driving are, however, sparse.

As far as is known to us, only Volkerts et al. (1992) conducted such a study. In this study, the residual effects of nocturnal treatments with hypnotics (i.e. lormetazepam 1 mg and oxazepam 50 mg) on simulated driving performance were compared to on-the-road driving performance. They found that the main parameter in the on-the-road task (SDLP) was sensitive to the verum, whereas simulated tracking performance was not. Volkerts et al. (1992) concluded that this difference was due to a lack of sensitivity of the driving simulator but proposed an alternative explanation as well. The simulator task was a curve following task, inducing a relatively high state of vigilance, which was more closely related to city driving, whereas the on-the-road driving test was a simple motorway driving task, inducing a relatively low vigilance state. The difference in drug effects could therefore also be attributed to a difference in task demands. Therefore, the primary aim of the present study was to investigate the sensitivity of the driving simulator for detecting drug effects in equally demanding driving tasks.

As a case in point, we investigated the dose-related effects of two dosages of oral cannabis (dronabinol; 10 and 20 mg) and placebo on driving performance. Testing the effects of ∆^9^-tetrahydrocannabinol (THC) on driving performance is important because the use of cannabis (in which THC is the primary active compound) is widespread throughout the world, and it is the most frequently detected illicit drug found in impaired drivers (Hartman and Heustis 2013). And although the influence of THC on driving performance has been tested before, both on the road in actual traffic (Ramaekers et al. 2000; Robbe 1994, 1998; Bosker et al. 2012a, b) and in the driving simulator (Lenné et al. 2010; Liguori et al. 2002; Ménétrey et al. 2005; Sexton et al. 2000), outcomes are difficult to compare because experimental setups and performance measures are usually dissimilar.

In the current study, we therefore tested in a within-subjects design whether driving simulator and the on-the-road driving tests were equally sensitive to detect dose-related effects of dronabinol on driving performance. Performance measures that were used are: the road tracking test as developed by O’Hanlon et al. (1982) and the car following test adapted from Brookhuis et al. (1994). Both tests have been used in numerous studies testing the effects of psychoactive substances on driving performance such as: alcohol (Veldstra et al. 2011; Mets et al. 2011), amphetamines (Simons et al. 2011), MDMA (Bosker et al. 2012a; Kuypers et al. 2006; Veldstra et al. 2011), hypnotics (Leufkens et al. 2007), anti-depressants (Wingen et al. 2006) and anti-histamines (Ramaekers and O’Hanlon 1994). Moreover, the main performance measure in the road tracking task, SDLP, has been calibrated on the road for alcohol-induced performance effects in such a way that clinically relevant performance increments can be expressed in equivalent BACs (Louwerens et al. 1987) using so-called equivalence tests.

The present work was part of a larger study on the effects of dronabinol on driving performance (Bosker et al. 2012a, b). In this paper, we tested whether the simulator and the on-the-road driving tests generated equal results in terms of clinical relevance.

## Methods and materials

### Participants

Twenty-four participants (14 male, 10 female) with an average age of 23.6 years (SD = 3.0) participated in a study on the effects of dronabinol on driving performance (for details, see Bosker et al. 2012b). Participants were experienced drivers who were in the possession of a valid driving licence over 3 years with a minimum mileage of 5000 km per year. This study was conducted in accord with the code of ethics on human experimentation established by the Declaration of Helsinki (latest revision, Seoul 2008) and in accordance with the Medical Research Involving Human Subjects Act (WMO). Approval for the studies was obtained from the Maastricht Academic Hospital’s Medical Ethics committee. A written informed consent was obtained from every participant. Participants were compensated for their participation by means of a monetary contribution and were driven home at the end of each testing day.

### Study design and treatment

The study was conducted according to a double-blind, placebo-controlled, crossover design with treatment orders but not driving conditions counter-balanced. The treatments consisted of a single dose of dronabinol (10 and 20 mg) and placebo. Administration occurred orally in identically appearing capsules.

### Procedure

When there was no medical objection for participation, participants were invited to come for a training day in which the participants practised the driving tasks and were asked some questions regarding feelings nausea, dizziness and headache after driving in the simulator to check for simulator sickness (i.e. a sort of motion sickness that can be experienced when driving in a simulator; see for example Stoner et al. (2012) in Fisher et al. (2011). After the training day, participants visited the facilities three times (1 day for each condition), with a washout period of at least 4 days in between. Participants were asked to refrain from the use alcohol on the day prior to a testing day and were requested to arrive at experimental sessions well rested (participants were asked if they had had enough sleep the day before testing).

Drug (morphine, cocaine, marijuana, methamphetamine and amphetamine) and alcohol screens were performed prior to experimental sessions upon arrival. Women were also tested for pregnancy. If the outcome of the test allowed the participant to proceed with the experiment, he/she was administered with 10 mg, 20 mg or placebo dronabinol. On-the-road driving tests were performed between 2 and 4 h after drug administration, and simulator driving tests were performed between 4 and 5 h postdrug administration. Two participants were tested per testing day. Participants first drove on the road for 1 h and then in the simulator.

### Simulator driving

Test rides were conducted in a (fixed-base) ST Software driving simulator consisting of a mock-up car with original controls (three pedals, clutch, steering wheel, safety belt, indicator and hand brake) linked to a dedicated graphics computer, registering driver behaviour, while the road environment and dynamic traffic are computed at 30 Hz+. Participants had a 210° view of the road environment. Other vehicles in the simulated world interacted with each other and the simulator car autonomously and behaved according to hierarchically structured decision rules that are based on human driving behaviour (Van Wolffelaar and Van Winsum 1992).

On a rural road of approximately 30 km with a posted speed of 100 km/h, the road tracking task was conducted. The road consisted of a two-lane straight road with a normal traffic density. The main parameter was weaving as measured by SDLP.

In the virtual car following the test adapted from Brookhuis et al. (1994; see Veldstra et al. 2009), the participants were instructed to follow a lead car (posted speed 80 km/h) at a short but safe distance. The lead car was programmed to accelerate and decelerate within a randomly varied frequency between 0.025 and 0.05 Hz (i.e. a cycle of 20–40 s). Participant responses to the speed changes (between 60 and 80 km/h) of the lead car were measured by assessing the coherence (the extent to which the pattern of speed changes of the lead and follow car correspond), the gain (degree of over- or underreaction to speed changes of the lead car; when there is an overreaction the gain is larger than 1, while at an underreaction the gain is smaller than 1) and, most importantly, the time to speed adaptation (the reaction time of the following car as response to speed changes of the lead car).

### On-the-road driving

Participants operated a specially instrumented vehicle. The vehicle was equipped with an electro-optical device mounted at the rear back of the car to continuously measure lateral distance of the vehicle to the left lane line so as to measure SDLP in the road tracking task. The signal of the device was digitised at a rate of 4 Hz to an on-board computer disk and offline edited by removing all data segments that revealed signal loss, disturbance or occurrence of passing manoeuvres directly (see for example Bosker et al. 2012a).

An optical distance sensor (DME 2000) was placed in the grill of the instrumented car for measurements in the car following task. The sensor emitted laser signals in the direction of a reflection board mounted on the leading vehicles towing bracket. Distance was deduced from the time lapse between the transmission and receipt of the signal at the receiving end of the distance sensor. Velocity of the leading vehicle was transmitted via telemetry to the following vehicle and stored on a computer disk along with the velocity of the following vehicle and headway. Speed signals collected during manoeuvres entered a power spectral analysis for yielding phase delay between the vehicle’s velocities at the manoeuvre cycle frequency.

Both the road tracking task and the car following task were performed in the right-hand lane of a relatively straight two-lane motorway with a normal traffic density. In the road tracking task, participants drove approximately 1 h under the instruction to keep a constant speed of 95 km/h and to drive in the centre of the lane as well as possible. In the car following task, participants drove for approximately 25 min behind a lead vehicle while maintaining a constant distance. The lead vehicle was under an investigator’s control, who initiated each manoeuvre by activating a microprocessor-driven cruise control which was set to maintain a constant speed of approximately 100 km/h. Sinusoidal speed changes reached an amplitude of −10 % and fell within a frequency of 0.2 Hz (50 s). Responses to speed changes were assessed by deducting the coherence, gain and delay.

### Pharmacokinetic assessments

Blood samples (8 mL) were collected 1.5, 4.25 and 6 h after drug intake. The blood samples were centrifuged and serum was frozen at −20 °C until analysis. THC, 11-hydroxy THC (11-OH-THC) and nor-9-carboxy-THC (THCCOOH) concentrations were determined afterwards using solid-phase extraction and gas chromatography with mass spectrometric (GC-MS) detection with limits of detection/limits of quantification of 0.24/0.73, 0.11/0.26 and 0.98/2.99 ng/mL, respectively (Mauden et al. 2000). The sum of molar concentrations of THC and 11-OH-THC was used to evaluate any confounding effects of cannabinoid concentrations between on-the-road (OR) and simulated (SIM) driving.

### Data analysis

All statistical analyses were conducted by means of SPSS 16 for Windows. Averages were subjected to a general linear model (GLM) repeated measures analysis with test environment (two levels) and treatment (three levels) as within-subject factors. If Mauchly’s test indicated that the assumption of sphericity was violated, the degrees of freedom were corrected using the Greenhouse-Geisser correction.

As Godley et al. (2002) already pointed out, statistically non-significant results validate the driving simulator. However, a non-significant result is not necessarily an indication of a genuine absence of difference. Lack of power due to insufficient sample size, and therefore insufficient statistical power, could also account for the results. Due to this limitation, we need information relating not only to the question of whether or not an effect exists but also to the magnitude of this effect. This is typically accomplished by estimating the effect size (Rosnow and Rosenthal 2009). In this paper the *η*^2^_p_ was used to calculate the effect size for *F* tests and interpreted using the guidelines provided by Cohen (1988). When the difference between on-the-road driving and simulator driving was non-significant and the effect size could be considered too small to be meaningful (e.g. *η*^2^_p_ ≤ 0.01), correspondence in absolute terms could be claimed. Relative validity was established by comparing treatment effects for both driving conditions. If treatment effects were comparable, validity in relative terms could be claimed.

When evaluating the effect of a psychoactive substance on driving performance, SDLP is an important outcome measure. When evaluating the effects of drugs on performance, it is common to look at the treatment effect (e.g. the treatment SDLP effect minus placebo SDLP) and test for equivalence of the effect to alcohol-induced effects with a so-called equivalence test (Mascha and Sessler 2011). In this test, the equivalence of drug effects -i.e. the difference to the placebo- is compared to a criterion level that is established in an alcohol reference study. For the on-the-road study, the alcohol criterion level is based on outcomes generated in a study conducted by Louwerens et al. (1987) in which the influence of 0.5 permille alcohol on SDLP was tested on the road. For the equivalence test in simulator driving alcohol criterion outcomes reported on alcohol and simulator driving as reported by Veldstra et al. (2011) were used. Equivalence is tested by assessing if the preestablished criterion levels fall within the 95 % confidence interval of the drug effects. If this is the case, then the drug effect is considered to be clinically relevant (Mascha and Sessler 2011). Since this test is an important part of the assessment of the relevance of the effects found either by a driving simulator or while driving on the road, outcomes of equivalence tests were also compared in the current study.

## Results

### Pharmacokinetics

Average THC, 11-OH-THC and THCCOOH concentrations are displayed in Table 1. There was a significant treatment effect of THC (*F*_1.19_ = 14.30, *p* = 0.001). Also, there was a significant effect of time after dosing on THC in serum (*F* (2,19) = 6.59, *p* = 0. 016); THC in serum was at a maximum at 1.5 h after drug intake and decreased after that. There was no significant difference in sum of molar concentrations of THC and 11-OH-THC between the on-the-road driving test (OR = 2–4 post drug) and simulator driving test (SIM = 4–5 post drug; *F* (2,19) = 1.05, *p* = 0.38).

**Table 1:** Average (SE) THC, 11-OH-THC and THCCOOH concentrations in serum (μg/L) after dronabinol treatment 1.5, 4.25 and 6.0 h postdrug intake

Dronabinol treatment	Time postdrug (h)								
	1.5			4.25			6.0		
	THC	11-OH-THC	THCCOOH	THC	11-OH-THC	THCCOOH	THC	11-OH-THC	THCCOOH
10 mg	5.65 (1.79)	4.77 (0.62)	36.83 (6.45)	2.84 (1.79)	2.33 (0.62)	30.39 (6.47)	2.48 (1.79)	1.98 (0.62)	26.52 (6.47)
20 mg	7.52 (1.75)	6.25 (0.62)	43.71 (6.33)	3.74 (1.79)	3.50 (0.62)	37.39 (6.47)	4.20 (1.87)	3.49 (0.65)	41.38 (6.77)

### Road tracking

#### Absolute validity

As can be seen in Table 2, SDLP in the simulator was higher than on the road. However, this difference was non-significant and coincided with a very low effect size giving support for absolute validity.

**Table 2:** Average (SE) performance measures for road tracking and car following for both simulated (SIM) and on-the-road (OR) driving and all treatments

Driving task	Driving environment	Dronabinol treatment			ANOVA	
		Placebo	10 mg	20 mg	Driving environment	Driving environment × treatment
Road tracking						
SDLP (cm)	OR	19.58 (0.81)	20.99 (0.85)	22.05 (0.91)	*F* (1.19) = 0.15, *p* = 0.71, *η* ^2^_p_ = 0.008	*F* (1.55) = 0.23, *p* = 0.74, *η* ^2^_p_ = 0.01
	SIM	20.27 (0.87)	21.13 (1.07)	23. 27 (1.41)		
Car following						
Coherence	OR	0.93 (0.006)	0.91 (0.009)	0.92 (0.007)	*F* (1.10) = 1.94, *p* = 0.19, *η* ^2^_p_ = 0.19	*F* (2.20) = 1.21, *p* = 0.32, *η* ^2^_p_ = 0.11
	SIM	0.90 (0.02)	0.91 (0.01)	0.87 (0.02)		
Gain	OR	1.22 (0.03)	1.19 (0.03)	1.30 (0.05)	*F* (1.11) = 20.35, *p* = 0.01, *η* ^2^_p_ = 0.64	*F* (2.22) = 1.78, *p* = 0.19, *η* ^2^_p_ = 0.14
	SIM	1.01 (0.02)	1.02 (0.03)	0.99 (0.02)		
Delay^a^	OR	2.75 (0.17)	3.70 (0.26)	3,20 (0.35)	*F* (1.10) = 6.14, *p* = 0.03, *η* ^2^_p_ = 0.38	*F* (2.19) = 0.96, *p* = 0.40, *η* ^2^_p_ = 0.09
	SIM	2.29 (0.15)	2.41 (0.20)	2.73 (0.25)		

^a^Reaction time to speed changes of the lead car (s)

#### Relative validity

**Fig. 1:**
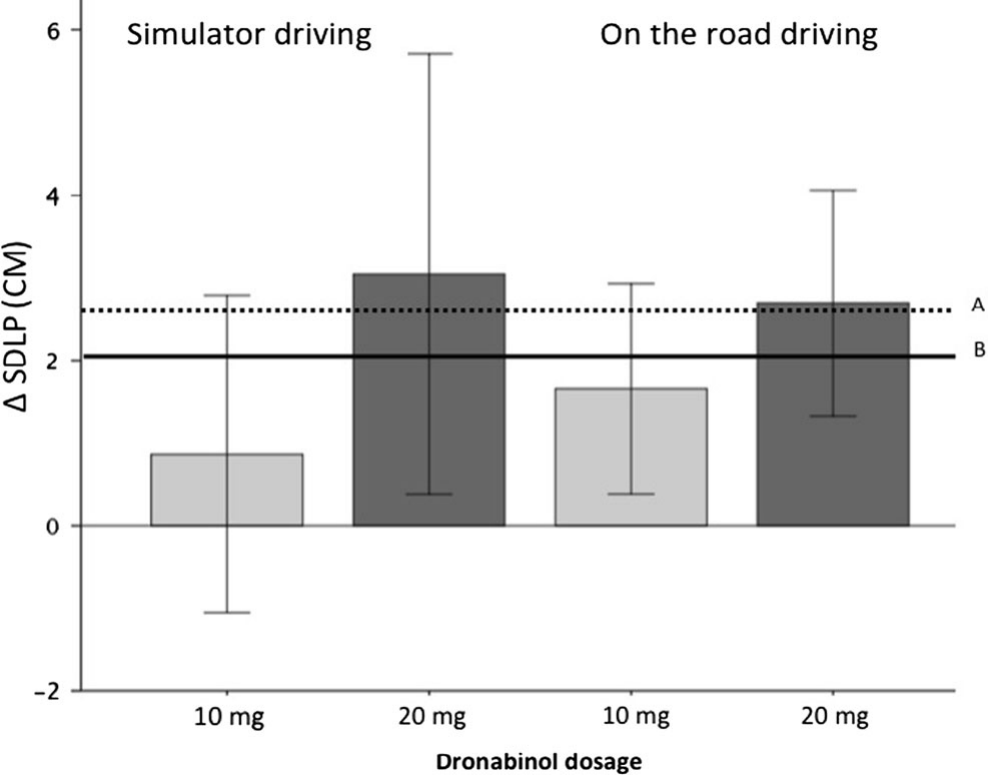
Average SDLP difference to placebo (95 % CI) as a function of dronabinol treatment when driving in the simulator versus on the road and alcohol reference lines: *A* 0.5 ‰ alcohol as measured on the road by Louwerens et al. (1987) and *B* 0.5 ‰ alcohol as measured in the simulator by Veldstra et al. (2011)

SDLP treatment effects (that is dronabinol minus placebo effect) differed non-significantly between driving conditions for both dosages (*F* (1.20) = 0.37, *p* = 0.55, *η*^2^_p_ = 0.02 and *F* (1.20) = .05, *p* = 0.83, *η*^2^_p_ = 0.002 for 10 and 20 mg, respectively). Effect sizes were very small for the 20-mg dronabinol treatment effect, but a bit higher for the 10-mg dronabinol treatment effect. There was no interaction effect for treatment × driving condition (see Table 2) indicating that treatment effects were not depended on driving condition. However, when analysing treatment effects for simulator and on-the-road driving separately, an important difference stood out. Although both driving conditions showed a significant main effect of dronabinol on SDLP (*F* (2.42) = 7.17, *p* = 0.002, *η*^2^_p_ = 0.26 and *F* (2.42) = 3.31, *p* = 0.05, *η*^2^_p_ = 0.14 for on-the-road driving and simulator driving, respectively), the strength of the treatment effect differed. In the on-the-road driving condition, both dosages differed significantly from placebo (*F* (1.21) = 6.31, *p* = 0.02, *η*^2^_p_ = 0.23 and *F* (1.21) = 14.69, *p* = 0.001, *η*^2^_p_ = 0.41 for 10 and 20 mg dronabinol, respectively), whereas in the simulator driving condition, only the highest treatment dosage of 20 mg dronabinol (*F* (1.21) = 4.94, *p* = 0.04, *η*^2^_p_ = 0.19) differed significantly from placebo and the lower dosage did not (10 mg: *F* (1.21) = 0.86, *p* = 0.36, *η*^2^_p_ = 0.04). This is also illustrated in Fig. 1.

The figure also illustrates that the 95 % confidence intervals that are used for equivalence testing seem to differ between the two testing conditions. The confidence intervals are wider and upper bounds are higher in the simulator driving condition as compared to on-the-road driving.

### Car following

#### Coherence: absolute validity

Comparing coherence in absolute terms showed no significant differences between driving conditions. Since the effect size was also smaller than the preset level of 0.01, absolute validity could be established.

#### Coherence: relative validity

There was no interaction effect for treatment × driving condition (see Table 2) indicating that treatment effects were not depended on driving condition. When analysing the treatment effects for both on-the-road driving and simulator driving separately, no treatment effect for both tasks (*F* (2.26) = 1.11, *p* = 0.34, *η*^2^_p_ = 0.08 and *F* (2.40) = 2.26, *p* = 0.12, *η*^2^_p_ = 0.10 for on-the-road and simulator coherence, respectively) were found, indicating relative correspondence.

#### Gain: absolute validity

The average gain as reaction to the lead car speed changes was significantly higher on the road than in the simulator (see Table 2) indicating that there was no correspondence between the driving conditions in absolute terms.

#### Gain: relative validity

There was no interaction effect for treatment × driving condition (see Table 2), and separate testing for on-the-road and simulator car following revealed that there was no treatment effect for both driving conditions (*F* (2.26) = 2.23, *p* = 0.12, *η*^2^_p_ = 0.15 and *F* (2.42) = 0.98, *p* = 0.38, *η*^2^_p_ = 0.05 for on-the-road and simulator gain, respectively).

#### Delay: absolute validity

The average reaction time to the speed changes of the lead car was higher on the road than in the simulator (see Table 2) indicating that there was no correspondence between the driving conditions in absolute terms.

#### Delay: relative validity

**Fig. 2:**
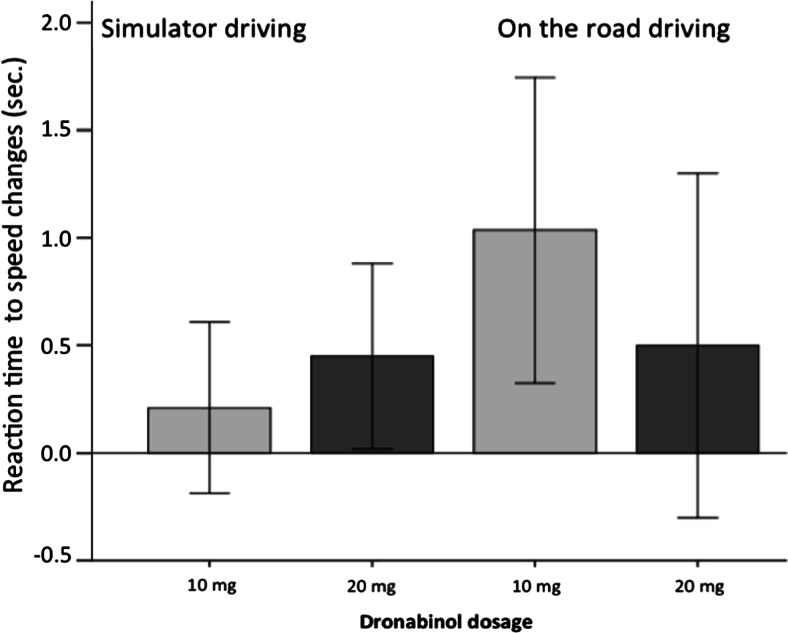
Average reaction time to speed changes (difference to placebo) and 95 % CI as a function of dronabinol treatment. Driving in the simulator versus on the road

Although there was no significant interaction effect, separate analyses of both driving conditions show differential effects of treatment on reaction time. There was a main treatment effect for dronabinol on reaction time when driving in the simulator (*F* (2.40) = 3.31, *p* = 0.05, *η*^2^_p_ = 0.14) but not for driving on the road (*F* (2.26) = 2.53, *p* = 30.10, *η*^2^_p_ = 0.16). When looking at Fig. 2, it becomes clear that reaction time to speed changes increased dose dependently when driving in the simulator, only reaching significance at the highest dosage (20 mg). However, on-the-road reaction time increased significantly in the 10-mg dronabinol condition but not for the 20-mg condition.

## Discussion

In the current study, we investigated whether or not the driving simulator was a sensitive instrument for assessing drug-induced effects on car driving by comparing effects of a drug on similar measures taken in the simulator and on the road. The tasks used to compare driving environments were the ‘road tracking task’ as devised by O’Hanlon et al. (1982) and the ‘car following task’ adapted from Brookhuis et al. (1994).

We used the two-threated approach to validity testing proposed by Blaauw (1982). This means that averages in the simulator and on the road were compared. If averages did not differ significantly, then the simulator was considered to be valid on an ‘absolute’ level of correspondence. If averages were influenced by an independent variable in the same way, then the simulator was considered to be valid on a ‘relative’ level of correspondence. In the case of the current research, the simulator was considered as valid on a relative level when the drug-induced effects that were found on the road were also seen in the simulator.

The results of the road tracking task showed that SDLP was comparable between driving conditions on an absolute level since averages did not significantly differ and the effect size of the main effect equalled the preset level of 0.01 (effect size considered too small to be meaningful). Also, the average treatment effects were not significantly different. Even so, there was a difference between on-the-road and simulator driving regarding the strength of the treatment effects. On the road, both treatment conditions significantly decreased performance on SDLP, but in the simulator, only the 20-mg dronabinol treatment effect had a statistically significant effect.

This lack of effect in the simulator for the lowest dronabinol dosage can be explained by a higher variance in the simulator than on the road as was illustrated by the difference in size of the 95 % confidence intervals around the treatment averages. It is relevant to find out if the higher variances in the simulator are due to random error or due to variance that can be attributed specifically to simulator driving since the upper bound of the confidence interval is used to test for equivalence of drug-induced impairments to alcohol-induced impairments so as to be able to assess clinical relevance of the results. In this case, for example, the higher upper bound of the 95 % confidence interval for the 20-mg treatment condition in the simulator would lead to more extreme conclusions about the possible impairing effects of the treatment on driving performance than equivalence tests for on-the-road driving data would.

Another possible explanation for not finding an effect at a lower dose of dronabinol on SDLP in the simulator compared to results found on the road is that not the driving environments but the drug concentrations differed. Even though previous research had shown that orally administered THC concentrations in blood peak after 1.5 h postdrug intake and then remain stable for about 4–5 h (Haney et al. 1999), average THC levels in serum were already decreased at 4.25 h postdrug intake. However, absolute differences in mean THC concentrations during on-the-road driving and simulated driving where minimal, i.e. 1 ng/mL after the 10-mg dose and about 4 ng/mL after the 20-mg dose. Therefore, differences in THC concentration may have caused some additional variance but are not likely to fully account for differential effects of dronabinol 10 mg during actual and simulated driving tests.

Car following results were less clear in terms of comparability. Coherence was comparable between driving conditions but phase and delay were not. Coherence was high and comparable in relative terms since both simulator and on-the-road driving condition showed no treatment effects. But also in absolute terms, coherence results were comparable since average treatment effects were non-significantly different—at least for the highest dose (20 mg dronabinol); the treatment effect of 10 mg dronabinol showed an effect size above the preset level of 0.01. Gain was not comparable in absolute terms since it was higher on the road than in the simulator, but was in relative terms since for both on-the-road driving and simulator driving, no treatment effect was found. For reaction to speed changes of the lead car, however, neither absolute nor relative correspondence was found. An increase in reaction time with dose was found when driving in the simulator, but not on the road.

These results indicate that participants were well able to follow the lead car in both the road and simulator driving conditions irrespective of dronabinol treatment. However, they tended to overreact to speed changes of the lead car more on the road as compared to in the simulator and had longer reaction times to speed changes of the lead car. These differences in car following performance in absolute terms are not problematic since the treatment effects of the car following task are generally not compared on an absolute level but in relative terms. However, since the reaction time to speed changes of the lead car was not comparable in relative terms either, one could conclude that car following results from simulator and on the road driving did not correspond.

It could be that differences in the way the car following task was presented on the road versus in the simulator influenced the outcome measures. For example, in the on-the-road car following task, participants were subjected to six to ten cycles of sinusoidal speed changes of the lead car in 25 min, whereas in the simulator car, the same amount of cycles were introduced in about 10 min of driving. The cycles in the simulator were also a bit shorter (i.e. 20–40 s) compared to on-the-road driving (50 s). Also, the posted speed in the simulator was lower (80 km/h) than on the road (100 km/h). Moreover, the simulator car following task is more controlled than the on-the-road driving task. Measurement disturbances such as other cars driving too slow in front of the lead car or cars merging between the lead and follow cars are not present in the simulator.

Also, the ability to estimate distance was different in the simulator versus on the road since the stereoscopic input differs between the two driving conditions. The simulator has a 2D vision on the car in front whereas in real life this is 3D. This might make it more difficult to perform this task in the simulator than on the road.

Another possible explanation is again that not the task but drug effects differed. However, this does not explain why a dose-related treatment effect of dronabinol on reaction time to speed changes of the lead car was found in the simulator and not or to a lesser extent on the road. The latter finding would actually argue for a better sensitivity of the simulator car following task in picking up drug-induced effects as compared to the on-road driving car following task.

### Limitations

For practical reasons, the on-the-road driving task always preceded the simulator driving task. This lack of counterbalancing may have influenced the outcomes. Furthermore, as explained, small differences in drug-induced effects may have occurred due to taking the simulator test somewhat later than the on-the-road driving test, which in turn may have affected the outcomes. Also, the investigated group was quite hetrogeneous since both occasional and regular cannabis users were included. This hetrogeneity of groups could have influenced the influence of the drug on performance somehow despite the within-subjects design.

Furthermore, factors that influence driving in both devices differentially could have influenced outcomes. Although the aim of the study was to investigate if outcomes of the two driving tasks (OR and SIM) could generate similar results irrespective of these factors, they are worth mentioning as potential limitations.

First, the devices can influence behaviour on a motivational level differentially. In the on-the-road driving task, for example, an instructor is sitting next to the participant, whereas in the simulator, there is no instructor present. This may prompt some participants to try and perform better for the instructor in the on-the-road driving task. On the other hand, the fact that one cannot have an accident in the simulator could influence motivation in the simulator differentially than while driving on the road. Although the chances of becoming involved in an accident are also nihil on the road since an instructor is there to intervene-, the percieved risk may be different i.e. driving in a real car may prime reactions that are associated with real driving while the simulator may not.

Second, the aforementioned difference in sensorial input between the simulator and on-the-road driving could also influence performance differentially since on-the-road driving occurs in a 3D and simulator driving in a 2D setting. Also, there is no tactical input in the simulator (which was fixed-based) while on the road there is. Also, for simulator driving participants can differ in their the susceptibility to simulator sickness, experience with driving in a simulator or gaming experience which can create extra variance that is not present in the on the road driving task. Another difference between the two devices is the controllability of traffic circumstances. In the simulator, everything can be controlled for, the weather, lighting, traffic flow etc., that cannot be controlled for on the road, leaving a gap between driving conditions on the road versus in the driving simulator.

For future research, it may be interesting to find out if the difference in variance between simulator and on-the-road driving that seemed to be present in this data is due to random error or due to some of the above-described factors in simulator driving.

### Conclusion

The driving simulator was proven to be sensitive for demonstrating dronabinol-induced effects particularly at higher doses. Treatment effects of dronabinol on SDLP were comparable with driving on the road but inter-individual variability seemed higher in the simulator than on the road which may have potential effects on the clinical inferences made from simulator driving. Car following on the road and in the simulator were, however, not comparable.
